# A crossover study of short burst oxygen therapy (SBOT) for the relief of exercise-induced breathlessness in severe COPD

**DOI:** 10.1186/1471-2466-11-23

**Published:** 2011-05-13

**Authors:** B Ronan O'Driscoll, Jane Neill, Siddiq Pulakal, Peter M Turkington

**Affiliations:** 1 Manchester Academic Health Science Centre University of Manchester Salford Royal University Hospital Stott Lane, Salford M6 8HD UK

## Abstract

**Background:**

Previous small studies suggested SBOT may be ineffective in relieving breathlessness after exercise in COPD.

**Methods:**

34 COPD patients with FEV1 <40% predicted and resting oxygen saturation ≥93% undertook an exercise step test 4 times. After exercise, patients were given 4 l/min of oxygen from a simple face mask, 4 l/min air from a face mask (single blind), air from a fan or no intervention.

**Results:**

Average oxygen saturation fell from 95.0% to 91.3% after exercise. The mean time to subjective recovery was 3.3 minutes with no difference between treatments. The mean Borg breathlessness score was 1.5/10 at rest, rising to 5.1/10 at the end of exercise (No breathlessness = 0, worst possible breathlessness = 10). Oxygen therapy had no discernable effect on Borg scores even for 14 patients who desaturated below 90%. 15 patients had no preferred treatment, 7 preferred oxygen, 6 preferred the fan, 3 preferred air via a mask and 3 preferred room air.

**Conclusions:**

This study provides no support for the idea that COPD patients who are not hypoxaemic at rest derive noticeable benefit from oxygen therapy after exercise. Use of air from a mask or from a fan had no apparent physiological or placebo effect.

## Background

Oxygen therapy is beneficial for many patients with chronic obstructive pulmonary disease (COPD). It is used to correct dangerous hypoxaemia in acute exacerbations of COPD and it is known that long term oxygen therapy (LTOT) can prolong life expectancy in patients with COPD who have got chronic hypoxaemia, especially if there is evidence of cor pulmonale [1-3]. It has also been demonstrated that the use of ambulatory oxygen therapy during exercise tests in the laboratory can increase exercise distance and reduce exercise-induced breathlessness in patients with COPD who desaturated during exertion although the reported benefits in the home environment were less impressive [4-7]. The most controversial form of oxygen therapy in COPD is known as short burst oxygen therapy (SBOT) [8]. This involves the use of oxygen either before or, more commonly, after exercise by patients with COPD who are not hypoxaemic at rest. This form of treatment appears to be especially common in the UK where it is estimated that about £18 million (approximately $25 million) per annum is spent on oxygen cylinders that are used in patients' homes [9] although there is very little evidence to support this practice. Previous short-term studies of SBOT for patients with COPD involved small numbers of patients and most of the trials failed to demonstrate any benefit from the use of SBOT either before or after exercise [10-19]. In one long-term study [20], patients who were randomized to "SBOT" using either oxygen or a placebo cylinder (air) over a six month period had high initial use of both types of cylinder and very low use subsequently and there was no difference between the use of oxygen or air cylinders. Two systematic reviews have concluded that the published studies do not support the use of SBOT for patients with COPD [8,21]. It has been suggested that some of the apparent benefits from oxygen therapy may be due to reflex mechanisms in response to a flow of cool air on the face or nose [22,23].

We conducted a partially single-blind study of oxygen compared with air from a face mask in patients with severe COPD who were not hypoxaemic at rest. Our aim was to determine if patients given oxygen would recover more quickly from exercise-induced breathlessness compared with patients given air. We also evaluated the possibility that patients might derive symptomatic benefit from having a flow of air delivered to the face by an electric fan.

## Methods

This trial was designed as a partially single-blind crossover assessment of the short-term response to SBOT amongst a group of patients attending the outpatient service in a University Hospital with severe COPD and limitation of activities of daily living due to breathlessness. The co-primary objectives were to determine whether SBOT given after exercise to non-hypoxaemic patients with COPD can (a) reduce dyspnoea during recovery from exercise or (b) shorten the recovery period. Oxygen therapy was compared with room air, compressed air from a face-mask or air blown on the face by an electric fan.

The secondary objectives were to determine; 1. Whether there is any difference in dyspnoea and time to recovery if a patient breathes room air, room air blown by an electric fan or compressed air via face mask; 2. Whether there is any difference in the subjective response to oxygen between patients who desaturate on exercise (SpO2 below 90% on at least two occasions during or after exertion in this study) and those who do not 3. Whether the subjective benefit from oxygen noted in some previous studies was due to a cooling air-flow on the face or a physiological benefit from Oxygen.

Patients were recruited from the chest clinic or pulmonary rehabilitation service at Salford Royal University Hospital. We recruited only patients who experienced significant breathlessness on minor exertion and who wished to be considered for symptomatic treatment with oxygen (SBOT) at home. Eligible patients were invited to take part in the study and given an information leaflet describing the study, with opportunities to ask questions of a doctor or respiratory nurse specialist prior to inclusion in the trial. Written informed consent was obtained before each patient could enter the study and the trial protocol was approved by the Salford and Trafford Local Research Ethics Committee, reference 04/Q1404/21.

### Inclusion Criteria

Patients aged over 50 years with smoking history of more than 20 pack years; Severe COPD with FEV1 less than 40% predicted value;[24] Oxygen saturation at rest ≥ 93% breathing room air at time of recruitment to study and on arrival on study day; Breathless during modest exercise such as climbing one or two flights of stairs; Stable for 4 weeks (Not requiring oral steroids or antibiotics or both); Able to give informed consent; Able to undertake a simple step test on an 18 cm exercise step.

### Exclusion Criteria

Unable or unwilling to give informed consent; Unable or unwilling to undertake step test for any reason; Complicating co-morbid conditions (e.g. arthritis of knees) which might interfere with exercise test or with the conduct of trial; Patients already using oxygen cylinders at home.

All exercise tasks were supervised by a single observer (JN). On the study day, patients completed the following procedures; The Oxygen saturation (SpO_2_) and baseline pulse were measured using a single Minolta Pulsox-3 oximeter. A Borg Dyspnoea score [25] (0 = not breathless, 10 = maximal breathlessness) was administered after at least 10 minutes of rest in a seated position. The patient then performed the first of four exercise step tests by stepping on and off a standard 18 cm exercise step as previously described [26,27]. Patients were instructed to exercise for up to 3 minutes at the fastest sustainable pace that was comfortable for them. The patients were instructed to stop before 3 minutes if they felt that they had reached comfortable limits (for whatever reason). The oxygen saturation and pulse rate were monitored continuously using the same finger oximeter for every test. The SpO2 and pulse rate were recorded in the patient's trial record sheet each minute during the exercise test, at the end of exercise and every minute during recovery until 5 minutes after finishing the exercise task. Patients completed a modified Borg dyspnoea score before and immediately after the exercise task and every minute for 5 minutes during the recovery period. The investigator recorded the objective recovery time defined as the time when the pulse rate had recovered to within five beats per minute of the baseline pulse. The subjective recovery time was measured by asking patients every minute during the recovery period if their sense of breathlessness had returned to the level that it was at before exercise.

Patients rested for at least 30 minutes after full recovery from each exercise task and they undertook a total of four exercise tasks on the same day. Immediately after completing each exercise task, patients received one of the following interventions in random order based on the patient drawing four pieces of folded paper sequentially from an opaque container prior to the commencement of the exercise task. Each piece of paper contained a single letter which determined the order of treatment as follows: a) Room air (no intervention). b) Use of an electric fan with 28 cm blades to blow cool air on the face from a distance of one meter (Type FT-30 fan from Guangdong Zhongshan Household Electric Appliance General Factory, China; set at highest speed setting). c) Air at 4 liters per minute from a simple face mask (Venticaire medium concentration oxygen mask, from Flexicare Medical Limited UK). *d) *Oxygen at 4 liters per minute (approximately 35% oxygen) from the same mask in a single blind manner. Allocation to treatment order took place after all other aspects of trial recruitment to avoid any possible recruitment bias. The oxygen and air treatments were single blind. To achieve blinding for these two interventions, the investigator connected a concealed air or oxygen cylinder delivering a gas flow of 4 liters per minute to the same face mask for each patient.

### Statistics

The study of Nandi et al [18] was used to determine the number of subjects required for the trial. We estimated that a crossover study involving 40 patients would have 80% power to detect a significant difference in the objective recovery time comparing oxygen with air. All data were entered in a scientific database (Graph Pad Prism4) and analyzed in that database. Data for each group were compared using Wilcoxon matched pairs test.

## Results

39 patients were recruited and gave written informed consent to take part in the study. Three patients were found to have recent FEV1 above 40% predicted and their results were not analyzed. One patient was hypoxaemic breathing air when he attended for the study (SpO_2 _88%) and one patient withdrew from the study after one of the four exercise tasks. Details of the 34 patients who completed the study are shown in Table 1.

**Table 1:** Details of 34 patients who completed the trial

	*Mean*	*Standard Deviation*	*Range*
**Age (years)**	67.5	8.9	52-87
**FEV1 (liters)**	0.80	0.2	0.45-1.3
**FEV1 as %predicted**	31.4%	5.5	21-39
**Number of steps climbed**	35.0	18.2	7-115
**Exercise time (seconds)**	93.9	43.1	23-180
**SpO2at rest**	95.0	1.3	92-98
**SpO2 at end of exercise**	91.3	3.8	79-97
**Pulse at rest**	86.1	12.4	51-117
** Pulse at end of exercise**	103	17.8	57-145
**Borg score at rest**	1.5	1.1	0-4
**Borg score at end of exercise**	5.1	1.6	2-9

(24 male, 10 female; 4 current smokers, 30 ex-smokers)

Patients climbed an average of 35 steps in an average of 94 seconds. The mean number of steps climbed (SD) was as follows. First task 34.9 steps (22.6); second task 35.1 steps (17.2); third task 35.2 steps (16.8); fourth task 35.0 steps (16.3). The patients' oxygen saturation and pulse rates were well matched before each exercise task and at the end of each of the four exercise periods as shown in table 2.

**Table 2:** Summary of responses to exercise and treatments.

	*Room Air*	*Electric Fan*	*Air Mask*	*Oxygen Mask*
**Mean exercise time (seconds)**	93.7 (42.1)	92.9 (43.2)	94.1 (40.5)	93.0 (46.1)
**Mean number of steps climbed**	34.1 (17.0)	36.0 (17.1)	36.6 (21.9)	33.4 (16.8)
**Mean pulse pre-exercise**	86.0 (12.1)	87.3 (13.1)	84.7 (12.0)	86.2 (12.6)
**Mean pulse at end of exercise**	99.3 (18.6)	103.6 (16.6)	107.0 (19.7)	102.1 (16.2)
**Mean SpO**_**2 **_**pre-exercise**	95.2 (1.4)	94.8 (1.3)	95.1 (1.3)	94.9 (1.3)
**Mean SpO**_**2 **_**at end of exercise**	91.3 (4.0)	91.1 (3.7)	91.3 (4.3)	91.5 (3.5)
**Mean Borg score pre-exercise**	1.5 (1.1)	1.5 (1.2)	1.5 (1.1)	1.6 (1.2)
**Mean Borg score at end of exercise**	5.1 (1.7)	5.1 (1.7)	5.3 (1.6)	5.1 (1.7)
**Mean subjective recovery time (*mins)***	3.2 (1.1)	3.6 (1.8)	3.3 (1.1)	3.1 (1.2)
**Mean objective recovery time *(mins)***	2.8 (2.0)	2.3 (1.1)	2.9 (2.5)	1.9 (1.0)
				
**Recovery time for sub-group of 14 patients who desaturated**				
**Mean subjective recovery time ***(mins)*	3.2 (1.1)	3.4 (1.1)	3.5 (0.9)	2.9 (1.2)
**Mean objective recovery time ***(mins)*	3.0 (1.8)	2.1 (0.9)	3.6 (3.6)	1.9 (1.0)

(Mean and SD)

**Figure 1:**
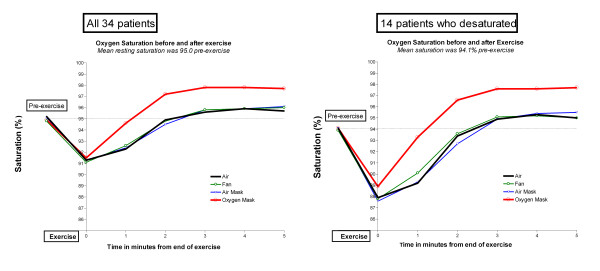
**Oxygen saturation before and after exercise**. Left hand panel: All 34 patients. Right hand panel: 14 patients who desaturated below 90%

**Figure 2:**
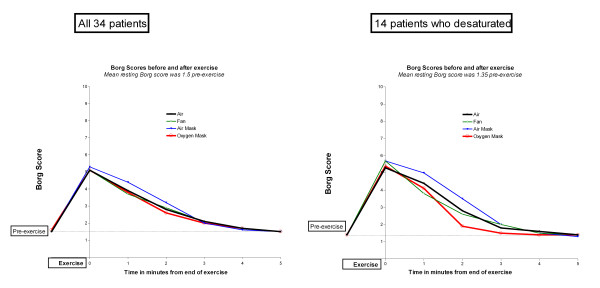
**Borg breathlessness scores before and after exercise**. Left hand panel: All 34 patients. Right hand panel: 14 patients who desaturated below 90%

**Figure 3:**
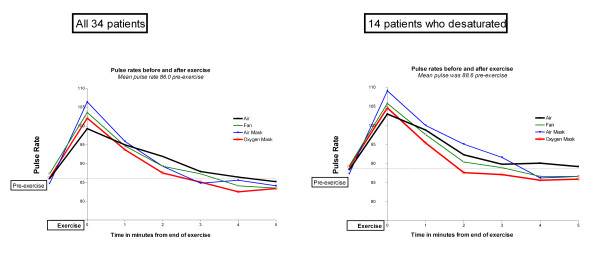
**Pulse rates before and after exercise**. Left hand panel: All 34 patients. Right hand panel: 14 patients who desaturated below 90%

The mean oxygen saturation fell from 95.0% pre-exercise to 91.3% at the end of the exercise task and the speed of SpO2 recovery to the baseline level was almost identical when room air, mask air and the electric fan were used in the recovery period (Figure 1). Oxygen saturation rose more quickly and to a higher level when the oxygen mask was used (p < 0.009 compared with the other treatments at 1 minute and <0.0001 thereafter). However, this increase in oxygen saturation of about 2% had no effect on the subjective sense of breathlessness as measured by the Borg score during the recovery period (Figure 2). The breathlessness score was about half a Borg unit higher when the air-mask was used compared with the other three groups at 1 and 2 minutes after finishing the exercise task (p = 0.01), possibly reflecting increased resistance due to the mask. Table 2 shows that the subjective recovery times were very similar for each of the four interventions with no significant difference between any of them. However, the pulse recovery time was almost a minute shorter when oxygen was given (Figure 3). This achieved statistical significance compared with room air (p < 0.01) and compared with air from a mask (p = 0.03) but not compared with air from a fan (p = 0.16).

The same pattern of oxygenation, Borg scores and recovery times was seen amongst the 14 patients who desaturated below 90% as was seen for the entire cohort with no significant difference in the Borg score at any time point or in the subjective recovery time compared with air. (Table 2 and right hand panels of Figures 1 and 2).

After completing the four exercise tasks, each patient was asked about their preferred treatment. Fifteen patients had no overall preference. Of the 19 patients who expressed a preference, 7 chose the oxygen mask, 6 chose the electric fan, 3 chose air from a mask and 3 preferred room air. The seven patients who expressed a preference for oxygen amounted to only 21% of the overall group. Five of these seven patients (71%) had desaturated during exercise compared with nine of 27 (33%) patients who did not have a preference for oxygen therapy but this difference did not reach statistical significance (Fisher's exact test p = 0.097).

## Discussion

Despite a rapid rise in oxygen saturation, COPD patients in this study reported no reduction in post-exertion breathlessness and no shortening of subjective recovery time when oxygen was given by simple face mask at 4 l/min compared with air from the same face mask or compared with a fan blowing air on the face or no intervention. This was true for the full group of 34 patients and for the sub-group of 14 patients who desaturated below 90% during exercise. The Borg score was about half a unit higher (worse breathlessness) when the air mask was used. Although this slight increase in breathlessness may have been caused by increased resistance to breathing due to the tight-fitting mask, the difference was probably too small to be clinically relevant. The pulse recovery time was shorter when oxygen was given but this did not seem to provide any symptomatic benefit. Quantrill [12] reported a small improvement of about 35 seconds in the subjective and objective recovery times when oxygen was given after exercise to patients with COPD who were already using SBOT at home and who reported benefit from it's use. However, these differences did not reach statistical significance and only 5 of 22 patients could correctly identify oxygen from air in Quantrill's single-blind study.

The present study was larger than all previously published studies of the effect of short burst oxygen therapy for the relief of breathlessness after exertion for COPD patients. The overall negative results are in line with the majority of previous studies [10-19] and with two systematic reviews of short burst oxygen therapy [8,21].

Some authors have suggested that cooling of the face or nose may reduce breathlessness by reflex actions [22,23]. However, we found that none of three interventions which involved gas flow over the face in the present study (air mask, oxygen mask or electric fan) had any significant effect on the patients' sensation of breathlessness after exertion. This suggests that any substantial physiological effect (or placebo effect) from airflow from masks or fans on the face is unlikely.

The Borg breathlessness scores were higher at one and two minutes post exercise when air was given by face mask at 4 l/minute (Figure 2). This effect was small but statistically significant (p < 0.01 compared with fan at one minute and compared with oxygen at two minutes and p < 0.04 compared with room air at one minute) and it was more marked for the 14 patients who desaturated (Figure 2, right hand panel). This might be due to the high respiratory rate in this group leading to a slight resistance to inspiration caused by inhaling from a small volume mask entraining a low flow of air [28].

Although the present study is not large, it is the largest laboratory study of SBOT yet conducted and the crossover design on a single day yielded very stable baseline values which made the comparison between treatments more robust. We did not achieve our recruitment target of 40 patients but the standard deviation of the Borg score post exercise (1.6) was lower than that the value of 1.8 which was reported in a previous similar study [19]. The achieved sample size of 34 patients gave a power of 0.94 to detect a difference of 1 Borg unit with a probability of type 1 error of 0.05.

The exercise step test is not as well validated in COPD as the 6 minute walk test or the shuttle walk test [29,30]. However, this test has been validated in previous studies [26,27] and there was excellent reproducibility with no learning effect from the first to the fourth exercise task in the present study which would further validate this test. A further advantage of the step test is that it is similar to the sort of activity such as stair climbing for which patients actually use SBOT in clinical practice [12]. Furthermore, the step test can be carried out in one to three minutes within the consulting room as part of a routine consultation but few clinics have sufficient staff time or floor-space to permit the use of 6 minute walks or shuttle walk tests within a routine consultation.

We observed no order effect in the present study . It is not known if any other outcome measure would be superior to the Borg score but the lack of benefit in Borg scores was reflected in the finding that only seven of 34 patients preferred oxygen compared with the other interventions. Five of these seven patients had desaturated during exercise. Although this was not statistically significant it raises the possibility that a larger study of "desaturators" might identify a small group of patients who could gain benefit from short burst oxygen therapy.

The present trial is in agreement with the majority of previous studies [10-19] in finding no overall improvement in the mean Borg breathlessness score when oxygen was given after exercise, even for the 14 patients who had experienced desaturation during the exercise task and despite a rapid rise in SpO2 on oxygen therapy and a quickening of the pulse recovery time. As none of the previous studies have reported large improvements in breathlessness in response to SBOT, the addition of the present study to the previous studies strengthens the current consensus view [1,8,21] that there is no clinically important benefit for most users of SBOT. By contrast, there is strong evidence of benefit from pulmonary rehabilitation for patients with COPD [31] and we would recommend this as the preferred option for patients who remain breathless on exertion despite maximal medical treatment for COPD.

Although it remains possible that a minority of patients might benefit from short burst oxygen therapy, ambulatory oxygen therapy (to prevent desaturation during exercise) might be more effective for such patients but it is not widely used at present for patients who are not hypoxaemic at rest. The GOLD (Global initiative for chronic Obstructive Lung Disease) Guideline [32] does not make any recommendation for or against the use of SBOT for patients with COPD. The British National Institute for Health and Clinical Excellence (NICE) has recommended that short burst oxygen treatment should "*only be considered for episodes of severe breathlessness in patients with COPD not relieved by other treatments....and only if an improvement in breathlessness following therapy has been documented*."[1] Our study provides further support for this advice.

## Conclusions

This study provides no support for the idea that COPD patients who are not hypoxaemic at rest derive noticeable benefit from oxygen therapy after exercise. This negative result was in agreement with most previous short-term trials of SOBT in patients with COPD. Use of air from a mask or from a fan had no apparent physiological or placebo effect. We would suggest that SBOT should not be prescribed without a formal exercise assessment such as that described in the present study and it should be prescribed for home use only if a patient with severe COPD has desaturated on exertion with demonstrable improvement in breathlessness on oxygen compared with air in a single-blind study and the prescription should be continued only if there is consistent use of the SBOT treatment over a period of several months.

## Abbreviations

COPD: Chronic Obstructive Pulmonary Disease; LTOT: Long Term Oxygen Therapy; SBOT: Short Burst Oxygen Therapy.

## Competing interests

The authors declare that they have no competing interests.

## Authors' contributions

All authors were involved in the design of the study. JN and SP recruited patients and also undertook the trial interventions and data entry into a computer database. RO'D analysed the data and wrote the text of the paper and all authors were involved in proofreading and editing the paper prior to submission for publication. All authors read and approved the final manuscript.

## Author' information

SP now works as a consultant respiratory physician at the University Hospital of South Manchester, Manchester M23 9RT

## Pre-publication history

The pre-publication history for this paper can be accessed here:

http://www.biomedcentral.com/1471-2466/11/23/prepub
